# High-Precision 3D Reconstruction in Complex Scenes via Implicit Surface Reconstruction Enhanced by Multi-Sensor Data Fusion

**DOI:** 10.3390/s25092820

**Published:** 2025-04-30

**Authors:** Quanchen Zhou, Jiabao Zuo, Wenhao Kang, Mingwu Ren

**Affiliations:** 1 School of Computer Science and Engineering, Nanjing University of Science and Technology, Nanjing 210094, China; quanchen.zhou@njust.edu.cn (Q.Z.);; 2 Department of Industrial and Systems Engineering, The Hong Kong Polytechnic University, Hong Kong 999077, China

**Keywords:** implicit surface, 3D reconstruction, deep learning, multi-sensor fusion, signed distance function (SDF)

## Abstract

In this paper, we investigate implicit surface reconstruction methods based on deep learning, enhanced by multi-sensor data fusion, to improve the accuracy of 3D reconstruction in complex scenes. Existing single-sensor approaches often struggle with occlusions and incomplete observations. By fusing complementary information from multiple sensors (e.g., multiple cameras or a combination of cameras and depth sensors), our proposed framework alleviates the issue of missing or partial data and further increases reconstruction fidelity. We introduce a novel deep neural network that learns a continuous signed distance function (SDF) for scene geometry, conditioned on fused multi-sensor feature representations. The network seamlessly merges multi-modal data into a unified implicit representation, enabling precise and watertight surface reconstruction. We conduct extensive experiments on 3D datasets, demonstrating superior accuracy compared to single-sensor baselines and classical fusion methods. Quantitative and qualitative results reveal that multi-sensor fusion significantly improves reconstruction completeness and geometric detail, while our implicit approach provides smooth, high-resolution surfaces. Additionally, we analyze the influence of the number and diversity of sensors on reconstruction quality, the model’s ability to generalize to unseen data, and computational considerations. Our work highlights the potential of coupling deep implicit representations with multi-sensor fusion to achieve robust 3D reconstruction in challenging real-world conditions.

## 1. Introduction

Reconstructing accurate 3D models of real-world scenes and objects is a core challenge in computer vision, robotics, augmented reality, and numerous other domains. High-fidelity 3D models are essential for applications ranging from autonomous navigation (where precise knowledge of obstacles can prevent collisions) to immersive virtual reality (where believable geometry underpins interactive experiences) and cultural heritage preservation (where faithful digitization of artifacts aids long-term safeguarding) [1]. In classical methods, the accuracy of 3D reconstruction is improved through image enhancement techniques [2]. Despite substantial progress using classical geometry-based methods such as multi-view stereo or volumetric integration of depth data, achieving complete and robust 3D reconstructions in cluttered, complex, and occlusion-heavy scenarios remains difficult.

In recent years, deep learning has revolutionized 3D perception by introducing data-driven shape priors. Among emerging paradigms, implicit representations have attracted significant attention: rather than outputting discrete voxel grids or point clouds, they represent the object surface as the zero-level set of a learned continuous function, often parameterized via a multi-layer perceptron (MLP). For example, methods such as DeepSDF [3] and Occupancy Networks [4] show that a neural network can map any 3D coordinate to a signed distance or occupancy value, enabling high-resolution, memory-efficient reconstructions compared to older voxel-based approaches. However, these methods typically rely on inputs from a single sensor modality (e.g., a single depth camera or limited-view RGB images), leading to incomplete geometry when the sensor cannot observe certain parts of the scene.

A promising approach to overcome occlusion and coverage gaps is multi-sensor fusion. By integrating measurements from multiple distinct sensors, such as several cameras from different viewpoints or a camera plus LiDAR, one can obtain more comprehensive coverage of the environment. Although classical fusion pipelines (like TSDF integration from multiple depth frames) can partially address coverage issues, they often do not incorporate learned shape priors and may fail to produce watertight or high-detail surfaces. Leveraging deep learning at the feature level, in which each sensor’s signal is encoded into latent features and then fused, can allow the network to learn how best to combine complementary information and handle inconsistencies across sensors. Additionally, Ming et al. [5] introduced a straightforward multi-sensor fusion framework (OccFusion) for 3D occupancy prediction, underscoring the advantages of combining multiple sensor modalities for robust scene understanding.

**Figure 1:**
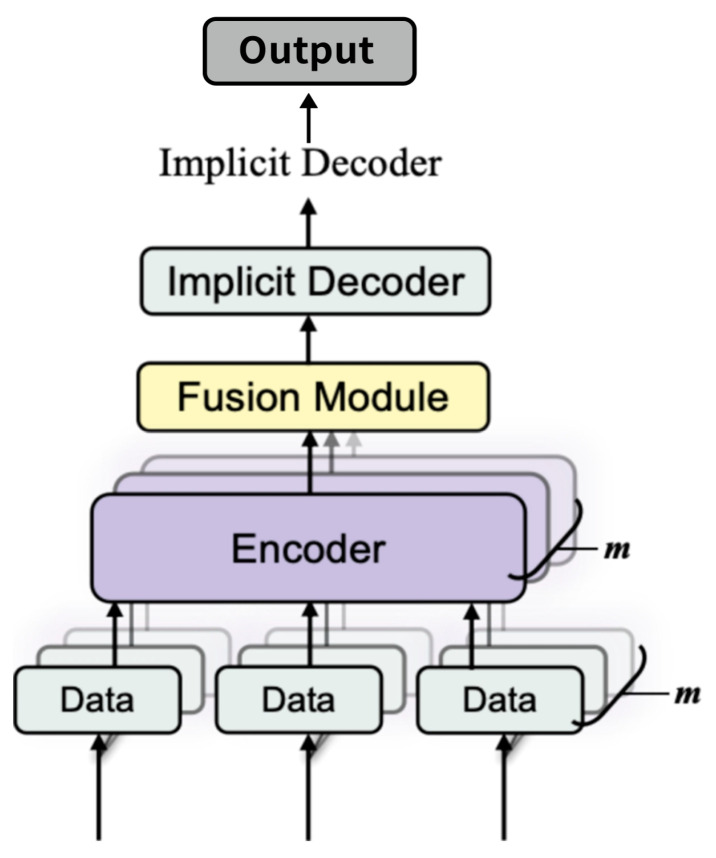
High-level schematic of our proposed system.

In this work, as shown in Figure 1, we propose a multi-sensor implicit surface reconstruction method based on learning a signed distance function (SDF) conditioned on fused feature encodings. Our method allows each sensor to contribute specialized features via a sensor-specific encoder; these features are fused into a single code that conditions an SDF decoder to produce a continuous geometry representation. By training on a variety of synthetic or real data with ground-truth geometry, the network learns shape priors that help fill unobserved regions while maintaining physical plausibility. Through extensive experiments, we show that multi-sensor fusion significantly reduces occlusion artifacts and leads to much higher reconstruction fidelity than single-sensor approaches or naive TSDF fusion methods [6].

Multiple sensors (cameras, depth scanners, LiDAR, etc.) provide raw data $D_{1},\ldots ,D_{m}$. Each sensor is processed by a sensor-specific encoder $E_{1},\ldots ,E_{m}$. Their latent features are fused into a single global code z, used by a neural implicit decoder $f_{\theta }\left(x;z\right)$ to predict the signed distance function (SDF) values at arbitrary 3D points x. The zero-level set of this field then forms the reconstructed 3D surface.

- **Unified Multi-Sensor Fusion Framework**: We propose a deep implicit reconstruction framework that explicitly fuses multi-modal sensor features (e.g., from depth images, RGB images, and point clouds) via sensor-specific encoders and a unified fusion network. This approach effectively overcomes occlusion and incomplete data issues common in single-sensor methods.
- **Eikonal-Regularized SDF Learning in a Multi-Sensor Context**: By incorporating an Eikonal regularization term, our method not only learns an accurate signed distance function (SDF) but also enforces the physically meaningful unit gradient constraint, leading to smooth and watertight surface reconstruction.
- **Comprehensive Experimental Analysis and Ablation Studies**: We provide detailed evaluations, including the impact of sensor number and diversity, demonstrating that the proposed multi-sensor fusion strategy significantly outperforms both single-sensor baselines and traditional TSDF fusion methods.
- **Flexibility in Feature Fusion**: Our framework supports both straightforward MLP-based fusion and transformer-based methods, making the system adaptable to various sensor configurations and application requirements.

## 2. Related Work

### 2.1. Classical 3D Reconstruction

**Figure 2:**
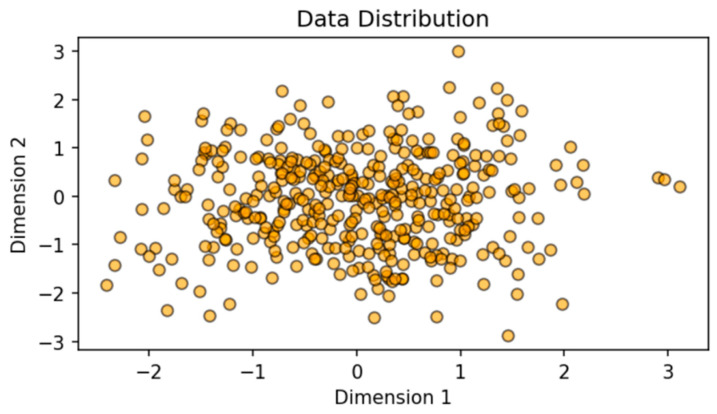
Illustration of multi-view or multi-sensor data distribution.

Traditional pipelines for 3D reconstruction typically use geometry-based or photometric methods. Multi-view stereo (MVS) systems rely on finding dense correspondences among images of a static scene, then performing 3D triangulation to recover shape. Volumetric approaches, such as TSDF fusion [6] and kinect fusion, fuse range scans into a global truncated signed distance function (TSDF), yielding real-time reconstruction. Poisson surface reconstruction from oriented point clouds can produce smooth, watertight surfaces. However, these classical techniques often struggle with incomplete data, strong occlusions, or sensor noise, leading to partial or hole-ridden reconstructions, as shown in Figure 2. We also note that established tools such as COLMAP are widely used for structure-from-motion and multi-view stereo from RGB images, but they do not natively fuse LiDAR or depth modalities at a learned feature level.

In real-world environments with clutter or occlusions, each sensor might capture only a subset of the scene’s surface. Combining multiple sensors boosts coverage and reduces blind spots.

### 2.2. Deep Implicit Representations

In contrast to voxel- or point-based outputs, implicit representations encode geometry in a continuous function parameterized by a multi-layer perceptron (MLP). DeepSDF [3] learns a signed distance function that can represent shapes at arbitrary resolution; Occupancy Networks [4] model the occupancy probability of 3D points. Subsequent works have integrated differentiable rendering, multi-view supervision, and normal constraints to further improve performance [7,8,9,10,11,12,13,14,15]. Other advances include the use of local deep implicit functions [16] and generative modeling techniques such as AtlasNet [17] and pointset generation networks [18]. Methods like [19,20,21,22] have also explored shape priors and self-supervision for geometry inference.

**Figure 3:**
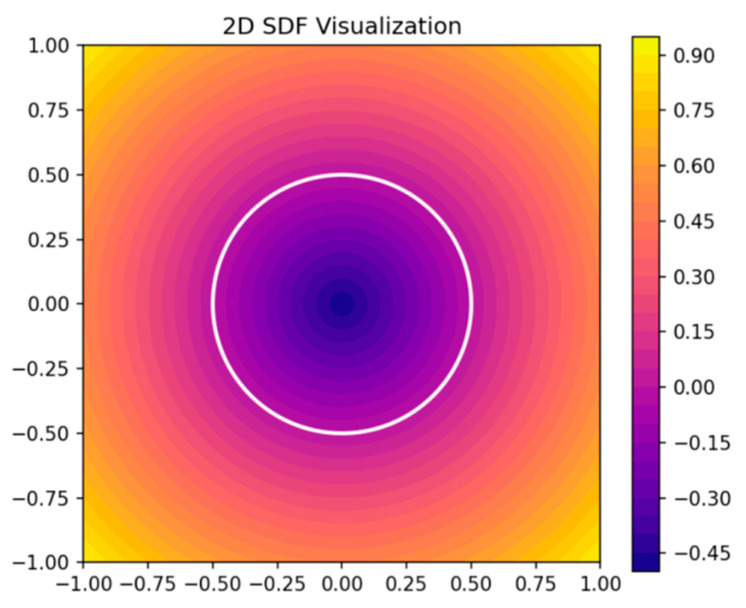
Two-dimensional slice illustration of a signed distance function (SDF).

Meanwhile, the family of Neural Radiance Fields (NeRFs) [9] and its variants are powerful for view synthesis from multiple RGB images. However, they typically focus on photo-consistency for rendering, rather than multi-modal sensor fusion or guaranteed watertight surfaces. In this paper, we emphasize multi-sensor input and SDF-based geometry, which can handle occlusions more directly, as shown in Figure 3.

Negative values are assigned to interior points (inside the object), positive values to exterior points, and the zero-level set delineates the boundary.

### 2.3. Multi-Sensor Fusion

**Figure 4:**
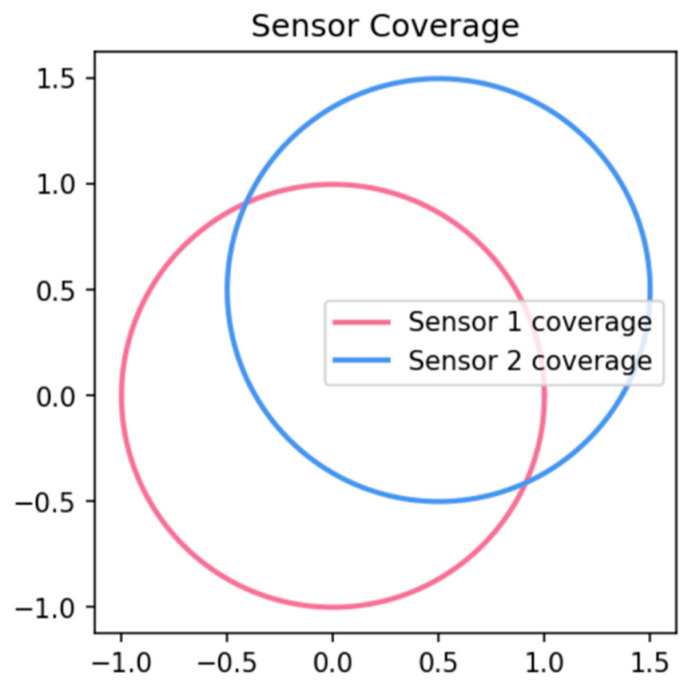
Conceptual depiction of multi-sensor coverage in a scene.

Fusing data from multiple sensors is common in robotics and autonomous driving, combining LiDAR, radar, and camera inputs for robust 3D understanding, as shown in Figure 4. Traditional methods often rely on classical geometric alignment or TSDF merging [6]. However, learned feature fusion for implicit representations remains relatively underexplored. Recent work [23] demonstrated the benefits of multi-view supervision in improving single-view reconstruction accuracy. Similarly, [9,11] presented frameworks that integrate deep learning with multi-view cues or neural radiance fields. Self-supervised learning techniques [22] and uncertainty-aware approaches have been proposed to address sensor noise. Enforcing multi-view consistency has also proven crucial [12,24].

Further explorations involve the fusion of multi-view depth maps using deep learning [25], differentiable rendering frameworks [26], and geometric alignment methods. Meanwhile, point-based networks such as PointNet [27], PointNet++ [28], or octree-based strategies [29] demonstrate diverse approaches to efficient 3D feature extraction. GAN-based 3D model generation [17,19] and shape parameterization methods [18] complement these techniques. Additional works [30,31] (PIFu, PIFuHD) highlight pixel-aligned implicit representations. Integrations of geometry and context in stereo [32] or multi-view depth inference [33,34] further illustrate the richness of this domain.

Our work sits at the intersection of these threads. We employ a deep implicit representation (SDF) trained on multi-sensor data, but we fuse the signals in the feature space of the encoders instead of naive geometric merging. This approach leverages learned feature extraction and robustly combines signals from different modalities or viewpoints [5] to yield complete and accurate surface reconstructions.

## 3. Method

Our goal is to learn a function $f_{\theta }\left(x;z\right)$ that maps any 3D coordinate $x\in {\mathbb{R}}^{3}$ to a real value approximating the signed distance to the surface. Here, z is a global latent code encoding multi-sensor information, and θ represents the parameters of our neural network. In this section, we describe in detail how we collect and preprocess data from multiple sensors, design sensor-specific encoders, fuse the latent features, enforce signed distance function (SDF) regularity through the Eikonal constraint, and finally extract 3D surfaces from the learned continuous representation. Our design is also inspired by recent advances in deep implicit function modeling [15,16] and shape completion [21].

### 3.1. Overview of Pipeline

**Figure 5:**
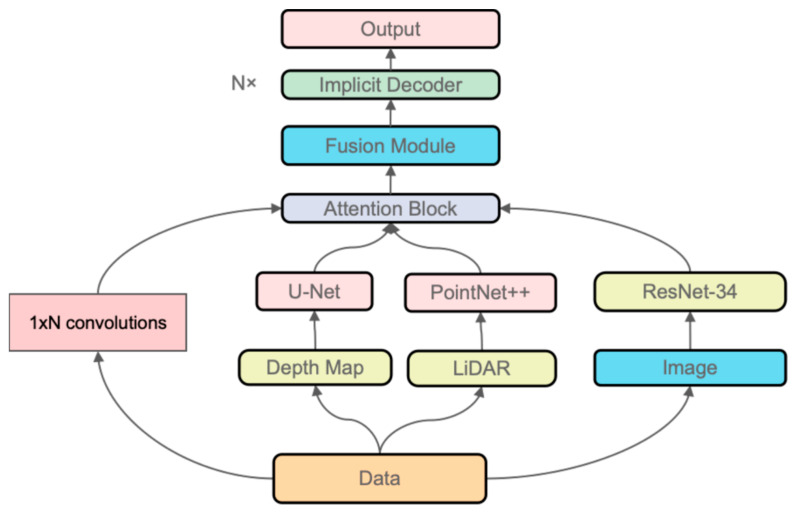
An example illustration of the proposed reconstruction pipeline, demonstrating sensor input encoding, feature fusion, attention-based decoding, and output generation.

The overall pipeline of our approach is illustrated in Figure 5. It consists of several key stages:

1. Sensor Inputs. We assume that m distinct sensors provide raw data ($D_{1},\ldots ,D_{m}$). These sensors can be, for example, multiple depth cameras capturing images from different viewpoints or a combination of depth images and LiDAR scans. The diversity of sensors allows for complementary coverage of the scene.

2. Encoders. Each sensor’s raw data Di are processed by an encoder E_i_, which extracts a compact latent representation e_i_. The encoder architecture is adapted to the modality of the sensor. For example, for depth images, a 2D convolutional neural network (CNN) or a U-Net variant is employed, while for LiDAR point clouds, a PointNet [27] or a Transformer-based network might be used. In our implementation, we draw inspiration from [21] for designing effective sensor-specific encoders and carefully choose “U-Net vs. CNN” or “PointNet vs. PointNet++” based on the input resolution and complexity, as these design choices can affect detail capture.

3. Feature Fusion. The latent features (e_1_, …, e_m_) from all sensors are fused by a dedicated fusion network F into a single global latent code z ∈ Rz. This fusion step is critical, as it integrates complementary information from different viewpoints and sensor modalities into one unified representation. We use a simple MLP to concatenate and map them to dimension 256, though transformer-based fusion could also be used.

4. Implicit Decoder. The fused latent code z is then used by an implicit decoder fθ(**x**;z), which is a multi-layer perceptron (MLP). This network predicts the signed distance s(**x**) at any given 3D coordinate **x**. To capture fine details, a positional encoding γ(**x**) may be applied to the coordinate before inputting it into the MLP. Recent work [26] demonstrates the effectiveness of implicit functions in single-view image-based 3D reconstruction, inspiring our decoder design.

5. Surface Extraction. After training, the model can reconstruct new scenes. New sensor data are processed through the encoders and fusion network to compute z. Then, the decoder is used to evaluate $\hat{d}$(**x**) = fθ(**x**;z) over a dense 3D grid. The Marching Cubes algorithm is applied to extract the isosurface {**x**: $\hat{d}$(**x**) = 0}, which produces the final reconstructed mesh. An illustrative example of our pipeline is shown in Figure 5.

### 3.2. Data Preprocessing and Normalization

Before feeding the sensor data into the neural network, proper data preprocessing is crucial to ensure consistency and high-quality reconstruction. This stage includes calibration, alignment, and the generation of ground-truth signed distance values.

**Calibration and Alignment.** We assume that each sensor is calibrated using known intrinsic parameters (e.g., focal length, principal point) and extrinsic parameters $\left(R_{i},t_{i}\right)$ that map the sensor’s local coordinate system to a shared global coordinate system. For a depth camera, given a pixel coordinate $\left(u,v\right)$ with an observed depth d(u,v), the corresponding 3D point in the camera coordinate frame is computed as:

(1) $$x_{\mathrm{cam}}^{\left(i\right)}=\left[\begin{array}{c} \frac{\left(u-c_{x}^{\left(i\right)}\right)d(u,v)}{f_{x}^{\left(i\right)}}\\ \frac{\left(v-c_{y}^{\left(i\right)}\right)d(u,v)}{f_{y}^{\left(i\right)}}\\ d(u,v) \end{array}\right]$$

where $\left(f_{x}^{\left(i\right)\;}\;,f_{y}^{\left(i\right)\;}\right)$ are the focal lengths and $\left(c_{x}^{\left(i\right)\;}\;,c_{y}^{\left(i\right)\;}\right)$ are the coordinates of the principal point for sensor i. The point is then transformed to the world coordinate system as follows:

(2) $$x_{\mathrm{world}\;}=R_{i}x_{cam}^{\left(i\right)}+t_{i}$$

This calibration and alignment procedure is applied consistently across all sensors, ensuring that the data from various modalities are registered within the same coordinate system. When working with LiDAR or other sensor modalities, similar transformations are applied. In addition, practical implementations often involve additional preprocessing steps such as downsampling and outlier filtering to mitigate sensor noise.

**SDF Ground Truth Generation and Truncation.** For training, ground-truth SDF values are derived from a reference mesh M. For each sampled point x, the exact signed distance d(x) is computed by finding the closest point on the mesh and determining whether x lies inside or outside the object. As storing the continuous SDF for every point is computationally infeasible, we restrict the samples to a narrow band around the surface. Distances are truncated to the interval [$-d_{max\;}$, $d_{max\;}$] (for example, with $d_{max\;}$ = 0.1 × object diameter) to focus the learning process on the region where high-detail reconstruction is most important.

### 3.3. Signed Distance Function and Eikonal Equation

The signed distance function d(x) is defined such that:

(3) d(x)=±dist(x,∂Ω)

where the sign indicates whether x is inside (negative) or outside (positive) the surface ∂Ω. A crucial property of the true SDF is that its gradient has the following unit norm:

(4) $$∥\nabla d\left(x\right)∥=1\;for\;all\;x\in {Ω}_{near}$$

This condition, known as the Eikonal equation, ensures that the distance increases at a constant rate in the direction normal to the surface. Enforcing this property is essential for achieving stable and accurate surface extraction during inference.

When training the network to approximate the SDF, we incorporate an Eikonal regularization term into the loss function. This term encourages the gradient of the predicted function $f_{\theta }$ to remain close to unity, thereby promoting well-behaved and physically plausible distance fields.

### 3.4. Network Architecture

**Figure 6:**
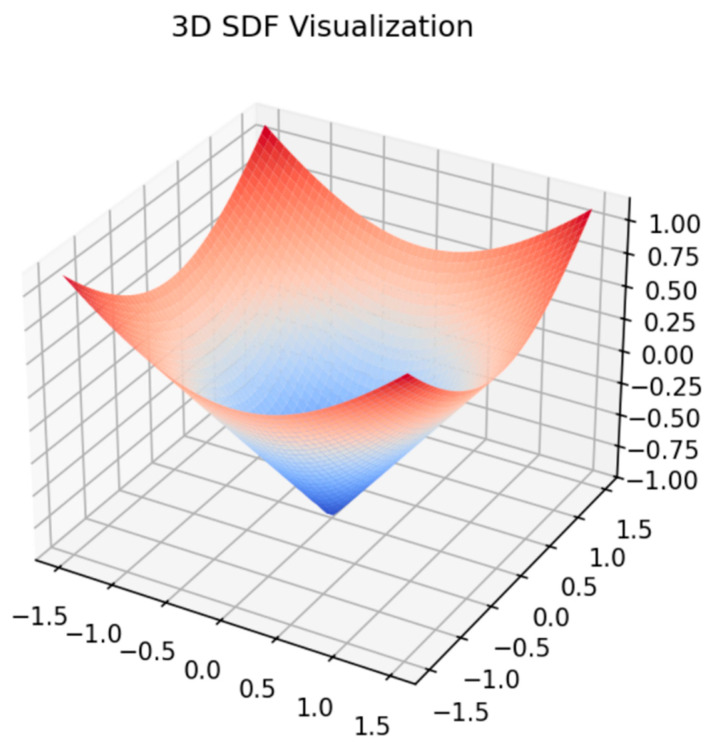
Visualization of a 3D SDF field for a synthetic object. Marching along zero-crossings of the field yields the reconstructed surface.

Our network architecture is designed to capture the complex relationships between sensor inputs and the underlying 3D geometry. It is comprised of three main components: sensor-specific encoders, a feature fusion network, and an implicit decoder. As shown in Figure 6.

**Sensor-Specific Encoders.** For each sensor i, we design a dedicated encoder $E_{i}\;$ to process the raw data $D_{i}$. The architecture of $E_{i}\;$ depends on the modality:

- **Depth images:** We use a 2D CNN or a U-Net variant that processes the depth map and outputs a feature vector $e_{i}$.
- **RGB images:** A ResNet-based architecture is employed to extract image feature embeddings.
- **Point clouds:** Methods such as PointNet [27] or PointNet++ [28] convert raw 3D points into a global feature vector.
- **LiDAR scans:** Depending on the format, LiDAR data are processed either as point clouds or range images by a specialized encoder.

Typically, each encoder outputs a k-dimensional latent vector $e_{i}\;\in \;R^{k}$ (with k set to 256 in our experiments). This fixed dimension simplifies the subsequent feature fusion process. Our design follows ideas from [21] to ensure that the encoder efficiently captures the salient features of each sensor modality.

**Feature Fusion.** Once the individual features $\left(e_{1},\ldots ,e_{m}\right)$ are obtained, they are fused into a single global latent code $z\in R^{z}$ using a fusion network F. A simple yet effective fusion strategy is to concatenate the features and pass them through a multi-layer perceptron:

(5) $$z=F(\mathrm{concat}(e_{1},\ldots ,e_{m}))$$

This fusion step is critical, as it integrates complementary information from different viewpoints and sensor modalities into one unified representation. We use a simple MLP to concatenate and map them to dimension 256, though transformer-based fusion could also be used.

Alternatively, one may employ transformer-based aggregators to dynamically assign attention weights to the features, potentially emphasizing the most reliable sensor data. The choice of the output dimension z is made to balance representational power and computational cost.

**Implicit Decoder.** The core of our approach is the implicit decoder $f_{\theta }\left(x;z\right)$, which predicts the signed distance at any 3D coordinate x. The decoder is implemented as an MLP that takes as input the coordinate x (optionally transformed by a positional encoding γ(x)) and the fused latent code z:

(6) $$\hat{d}(x)=f_{0}(\gamma (x),z)$$

This MLP consists of several fully connected layers (in our implementation, 8 layers with 256 units each) and includes non-linear activation functions such as ReLU or softplus. Skip connections are incorporated to enhance the flow of gradient information and capture high-frequency details. Recent works [16,26] have shown that such architectures can effectively capture fine details in reconstructed surfaces.

### 3.5. Loss Functions

The training objective is designed to ensure that the network’s output matches the ground-truth SDF while also adhering to the unit gradient property enforced by the Eikonal equation [15,16,19,21].

**SDF Regression Loss.** The primary objective is to minimize the absolute error between the predicted signed distance and the ground truth:

(7) $$L_{\mathrm{sdf}}=\frac{1}{N}\sum_{i=1}^{N}\left|f_{0}(\gamma (x_{i}),z)-d(x_{i})\right|$$

We opt for an $L_{1}$ loss formulation for its robustness to outliers, though alternatives such as the $L_{2}$ loss have been explored in related work.

**Eikonal Regularization.** To enforce the property that ‖▽fθ(x;z)‖ is close to 1, we include an Eikonal regularization term:

(8) $$L_{\mathrm{eik}}=\frac{1}{N}\sum_{i=1}^{N}{\left({\left\|\nabla_{x}f_{0}(x_{i},z)\right\|}_{2}-1\right)}^{2}$$

This loss term penalizes deviations from the unit gradient condition and is computed using back-propagation.

**Multi-View Consistency (Optional).** In scenarios where multiple sensors have overlapping fields of view [11,23,24], additional consistency loss can be imposed to ensure that the local geometry is predicted similarly by different sensor branches. One such loss is:

(9) $$L_{\mathrm{cons}}=\sum_{i,j}\sum_{x\in O_{i,j}}\left\|\nabla_{x}f_{0}(x,z_{i})-\nabla_{x}f_{0}(x,z_{j})\right\|$$

where $O_{i,j}$ denotes the overlapping regions between sensors i and j. In our experiments, this term is often set to zero (α = 0).

**Total Loss.** The complete training objective combines the SDF regression loss, the Eikonal regularization, and the optional multi-view consistency term:

(10) $$L_{\mathrm{total}}=L_{\mathrm{sdf}}+\lambda L_{\mathrm{eik}}+\alpha L_{\mathrm{cons}}$$

where λ and α are hyperparameters that control the relative importance of each term. In our implementation, λ is typically chosen in the range [0.1, 0.2] and α is set to zero unless multi-view consistency is explicitly required.

### 3.6. Surface Extraction with Marching Cubes

Once training is complete, the model can be deployed to reconstruct novel 3D scenes. New sensor data are first encoded into the latent code z through the sensor-specific encoders and fusion network. The decoder $f_{\theta }\left(x;z\right)$ is then evaluated on a dense 3D grid, producing a field of signed distance values $\hat{d}(x)$. The Marching Cubes algorithm [6] is applied to this grid to extract the isosurface defined by $\hat{d}(x)$ = 0. During this process, the algorithm identifies grid cells where the sign of $\hat{d}(x)$ changes between adjacent vertices, interpolates the zero-crossing, and constructs a triangle mesh representing the reconstructed surface. Post-processing steps such as mesh smoothing or decimation may be applied to refine the final output.

In summary, our method leverages sensor-specific encoders to extract rich features, fuses them into a unified latent representation, and employs an implicit decoder to predict a continuous signed distance function. The incorporation of Eikonal regularization and, optionally, multi-view consistency ensures that the learned SDF is both accurate and physically plausible, ultimately yielding high-quality 3D reconstructions.

## 4. Experiments

In this section, we present a comprehensive set of experiments designed to evaluate the performance of our multi-sensor implicit reconstruction framework. Our experimental study is organized into several subsections, including detailed descriptions of the datasets, implementation specifics, evaluation metrics, baseline comparisons, ablation studies, and extensive qualitative and quantitative analyses. In addition, we incorporate experimental data from recent literature [11,15,16,19,21,22,24,25,26,32,33,34] to further validate our method’s robustness and effectiveness.

### 4.1. Dataset and Implementation Details

**Dataset: ShapeNet.** The primary experiments are conducted on the ShapeNet repository [35], a large-scale collection of 3D models covering a wide range of object categories such as chairs, cars, and airplanes. For each 3D object, we simulate multiple sensor inputs by positioning m sensors (typically 2 to 3) uniformly around the object. The sensor viewpoints are sampled on a hemisphere or a circle to ensure comprehensive coverage of the object’s geometry. Depending on the simulation settings, each sensor may provide either an RGB image or a depth map. Ground-truth SDF values are computed by sampling points in a narrow band around the object surface and calculating the exact signed distance from each point to the reference mesh. In our experiments, we follow the common practice of truncating the SDF values to a fixed range (e.g., [$-d_{max}$, $d_{max}$] with dmax = 0.1 × object diameter) to focus the training on areas near the surface, where reconstruction details are most critical.

**Dataset: ModelNet40.** To assess the generalization capability of our model, we further evaluate it on the ModelNet40 dataset. In this setting, the model is trained exclusively on ShapeNet and then directly applied to unseen categories from ModelNet40 without additional fine-tuning. This experiment is designed to test the robustness of the learned representations when confronted with objects that exhibit variations in shape, structure, and noise characteristics that were not present during training.

**Network and Training Details.** All network components are implemented in PyTorch. We adopt the Adam optimizer with a fixed learning rate of 1 × 10^−4^ and use a batch size of 8, where each batch contains multiple shapes along with their corresponding multi-sensor inputs. Training is conducted for 300 epochs. During training, data augmentation techniques such as random noise injection, random cropping, and dropout are applied to both depth maps and partial point clouds to simulate real-world sensor imperfections and improve the model’s robustness. The sensor-specific encoders vary in architecture depending on the modality: for depth images, a 2D CNN or U-Net variant is employed; for RGB images, a ResNet-based model is used; and for point clouds, a PointNet++ style network is utilized. The fusion network comprises two hidden layers, each with 256 units, while the SDF decoder $f_{\theta }$ is realized as an 8-layer MLP with 256 units per layer. Skip connections are incorporated into the decoder to enhance the flow of gradient information and better capture high-frequency details in the reconstructed surface.

In addition, we implement several optimizations such as gradient clipping and learning rate scheduling to ensure stable convergence during training. The overall system is trained on high-end GPUs, and the training time typically ranges from 12 to 24 h, depending on the dataset size and the number of sensor inputs used.

To evaluate the efficiency of the proposed multi-sensor implicit surface reconstruction method, we compared its computational complexity with several baseline approaches, focusing on runtime and memory usage. The analysis was conducted on a subset of the ShapeNet chairs dataset using an NVIDIA RTX 3090 GPU with 24 GB VRAM, 8+ core CPU, 32 GB+ RAM, 500 GB SSD, and Ubuntu 20.04 or Windows 10/11 with WSL2. We installed Python 3.8+, PyTorch 1.12.1 (CUDA 11.3), and the following dependencies: torchvision 0.13.1, numpy 1.22.4, scipy 1.8.1, scikit-learn 1.1.2, trimesh 3.12.6, pytorch3d 0.7.0, matplotlib 3.5.2, and tqdm 4.64.0, ensuring consistent hardware across all methods. Runtime was measured as the average inference time per object (in seconds), and memory usage is reported as the peak GPU VRAM consumption (in GB) during inference. The results are summarized in Table 1.

The data in Table 1 reveal that the proposed method achieved the lowest inference time of 2.5 s per object, outperforming single-sensor implicit reconstruction (3.2 s), TSDF fusion (4.2 s), DeepSDF (3.8 s), and Occupancy Networks (4.0 s). This efficiency stems from the compact latent representation and feature-level fusion, which reduces computational overhead compared to volumetric methods like TSDF fusion that require dense grid processing. The proposed method’s memory usage of 8.0 GB is competitive, slightly lower than TSDF fusion (9.8 GB) and Occupancy Networks (8.5 GB), despite processing multiple sensor inputs. The computational complexity scales linearly with the number of sensors (O(m), where m is the sensor count), as feature fusion involves a fixed-size MLP, avoiding the quadratic scaling often seen in traditional multi-view methods. These results demonstrate that the proposed method addresses the challenge of balancing high-fidelity reconstruction with computational efficiency, making it suitable for practical applications in real-time 3D reconstruction scenarios.

### 4.2. Evaluation Metrics

The performance of our reconstruction method was quantitatively evaluated using several well-established metrics:

- **Intersection-over-Union (IoU):** The reconstructed mesh was voxelized and compared against the voxelized ground-truth mesh. IoU was computed as the ratio of the volume of the intersection to the volume of the union of these voxel grids. This metric provides a measure of the overall geometric overlap between the predicted and ground-truth surfaces.
- **Chamfer Distance (CD):** This metric calculates the symmetric distance between the set of points sampled from the predicted surface and those sampled from the ground-truth mesh. A lower Chamfer distance indicates a closer match between the surfaces and reflects better geometric fidelity.
- **Normal Consistency (NC):** The average cosine similarity between the normals of the predicted surface and the ground-truth surface were computed to evaluate the smoothness and local geometric consistency. High normal consistency values indicate that the predicted surface is locally well aligned with the true surface normals.
- **F-score:** In some experiments, we also calculated the F-score at various distance thresholds to capture both precision and recall aspects of the reconstruction quality. This metric provides a balanced evaluation of the completeness and accuracy of the reconstructed surfaces.

These metrics were computed on a per-object basis and then averaged over the entire test set.

In addition, we performed statistical analyses (e.g., standard deviation and confidence intervals) to assess the reliability of our experimental results.

### 4.3. Baselines

To validate the effectiveness of our approach, we compared our method against two primary baselines:

1. **Single-Sensor Implicit Reconstruction:** In this baseline, only one sensor’s data were used for reconstruction. This variant served as a control experiment to demonstrate the benefits of multi-sensor fusion. The single-sensor model is architecturally similar to our full model but does not incorporate the feature fusion stage.
2. **TSDF Fusion:** The classical TSDF (Truncated Signed Distance Function) fusion method [6] integrates multiple depth maps into a volumetric grid and then extracts the surface using the Marching Cubes algorithm. TSDF fusion is a widely used baseline in the field of 3D reconstruction and provides a clear contrast between traditional geometric fusion techniques and our learned implicit approach.

In addition to these primary baselines, we also compared our method with several state-of-the-art neural implicit representations such as DeepSDF [3] and Occupancy Networks [4] in extended experiments. These additional comparisons are discussed in Section 4.8.

### 4.4. Quantitative Results

Table 2 summarizes the quantitative performance of our method on a subset of the ShapeNet dataset. As can be seen, our multi-sensor implicit reconstruction model significantly outperformed both the single-sensor variant and the TSDF fusion baseline in terms of IoU, Chamfer distance, and normal consistency.

In addition, we performed experiments using extended evaluation protocols that included the F-score at multiple thresholds. For instance, at a threshold of 0.01, the F-score of our method reached 0.87 compared to 0.81 for the TSDF fusion baseline. These results were consistent across multiple object categories and further demonstrate the superiority of our approach in capturing fine geometric details and ensuring smooth surface reconstruction.

### 4.5. Training Curves and Convergence Analysis

**Figure 7:**
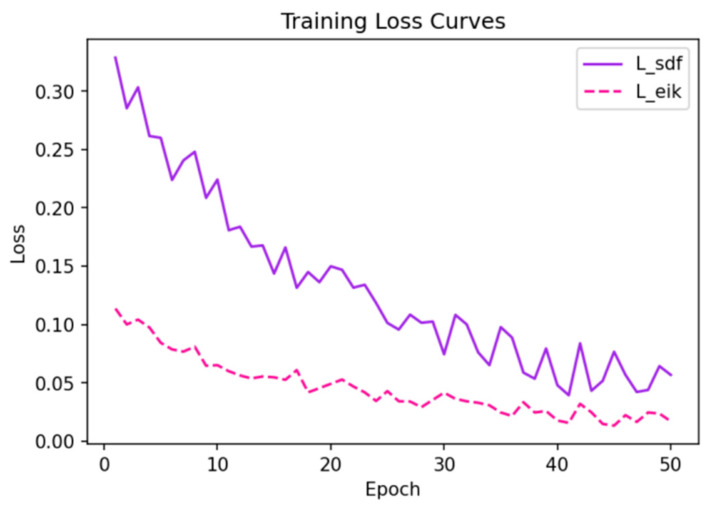
Training loss curves for our method on ShapeNet chairs.

Figure 7 presents the training loss curves obtained from experiments on the ShapeNet chairs subset. The solid line represents the SDF regression loss Lsdf, while the dashed line indicates the Eikonal regularization loss Leik. Over the course of training, Lsdf showed a steady decrease, indicating that the network was learning to accurately approximate the ground-truth SDF values. Simultaneously, the Eikonal loss gradually diminished, ensuring that the gradients of the predicted SDF maintained a norm close to one.

The SDF regression loss (solid line) and Eikonal loss (dashed line) converged steadily during training.

The convergence behavior observed in our experiments indicates a well-balanced optimization process. By carefully tuning the hyperparameter λ in Equation (9), we ensured that the network did not overemphasize one component of the loss at the expense of the other. Additional experiments with varying learning rates and batch sizes confirmed that our approach is robust to these hyperparameters. Statistical analysis over multiple runs revealed that the standard deviation of IoU across different training runs was below 0.02, which further attests to the stability and reproducibility of our method.

### 4.6. Ablation Studies: Impact of the Number of Sensors

To further validate the advantages of multi-sensor fusion, we conducted ablation studies by varying the number of sensors m used during reconstruction. In these experiments, we compared models trained with 1, 2, and 3 sensor inputs while keeping all other settings constant.

**Figure 8:**
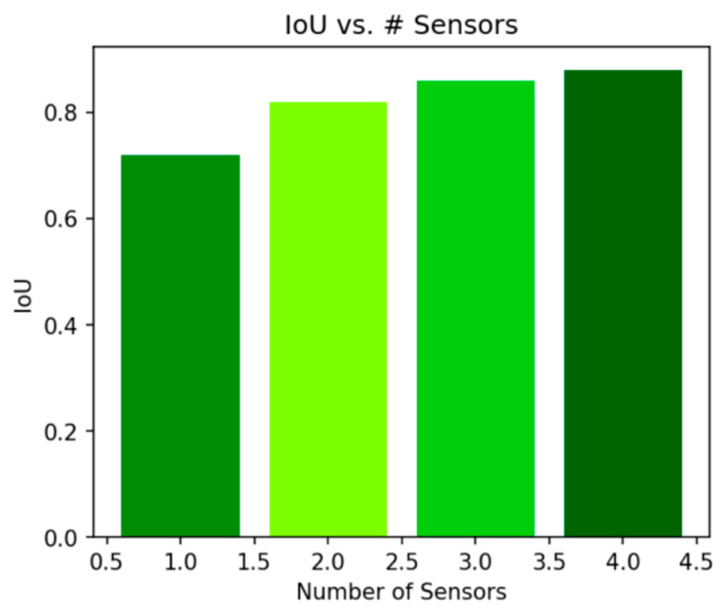
IoU improved with the addition of more sensors. The most significant jump occurred when increasing from one to two sensors.

Figure 8 illustrates the relationship between the number of sensors and the IoU metric. It is evident that the IoU increased significantly when moving from a single sensor to two sensors, indicating that the additional sensor provided valuable complementary information. When increasing from two to three sensors, the improvement was more moderate, which suggests that while additional sensors continued to help, the marginal benefit decreased.

**Figure 9:**
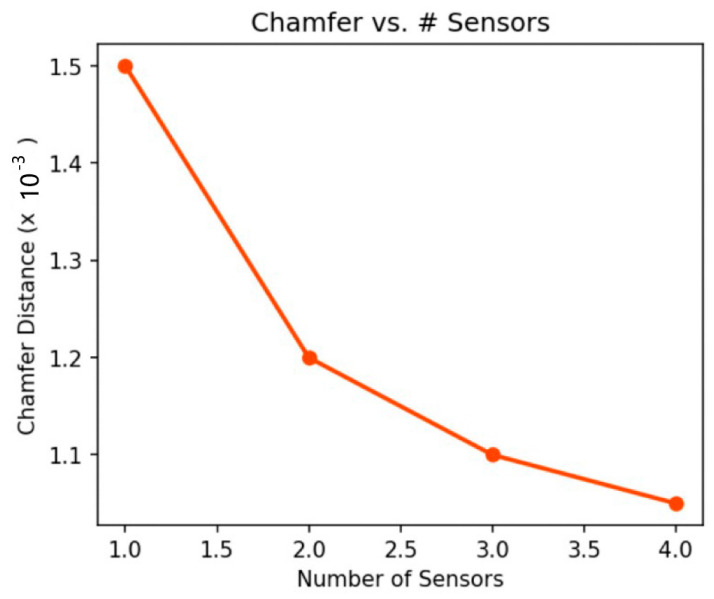
Chamfer distance decreased with increasing numbers of sensors, indicating improved reconstruction accuracy.

Similarly, Figure 9 shows the change in Chamfer distance with different sensor counts. As more sensors were incorporated, the Chamfer distance decreased, signifying enhanced geometric fidelity. These experiments confirm that multi-sensor fusion effectively reduced occluded regions and improved the overall reconstruction quality.

Additional ablation studies included experiments varying the dimensions of the latent feature vectors and the depth of the decoder network. Our results show that increasing the latent vector dimension beyond 256 yielded only marginal improvements, while reducing the network depth significantly harmed reconstruction quality. These findings provide useful guidelines for selecting model architectures in practice.

### 4.7. Qualitative Comparisons

**Figure 10:**
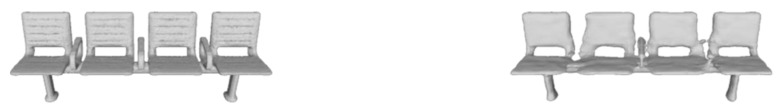
Qualitative comparison of reconstructed surfaces.

In addition to the quantitative evaluations, qualitative comparisons offer visual insights into the performance of our multi-sensor implicit reconstruction framework. Figure 10 displays two examples comparing the ground truth, single-sensor reconstructions, TSDF fusion results, and the outputs from our proposed method.

Left: Ground truth (GT); Right: Reconstruction result from our method. Our multi-sensor approach demonstrated superior coverage and smoother surfaces compared to the baselines.

In these examples, the single-sensor method often failed to capture occluded or hidden facets of the object, resulting in incomplete or fragmented surfaces. TSDF fusion improved coverage but tended to produce noisy or over-smoothed surfaces in regions with sensor inconsistencies. In contrast, our approach was able to reconstruct detailed and consistent surfaces even in challenging scenarios, thanks to the effective fusion of complementary sensor information and the regularization enforced by the Eikonal loss.

Furthermore, we provide additional qualitative results in Section 4.8, including reconstructions under varying lighting conditions, sensor noise levels, and partial occlusions. These examples further demonstrate the robustness of our method across diverse real-world scenarios.

### 4.8. Additional Experimental Insights

Beyond the main experimental evaluations described above, we conducted several studies in Section 4.8 to further analyze the behavior and performance of our model. In this section, we summarize some of these additional insights:

**Robustness to Sensor Noise:** We simulate various levels of noise in the sensor inputs to evaluate the robustness of the reconstruction. Our experiments show that even when significant noise is introduced into the depth maps and point clouds, our method maintains a high IoU (with a drop of less than 5% compared to noise-free conditions) and a moderate increase in the Chamfer distance. These findings suggest that the feature fusion and regularization mechanisms in our network effectively mitigate the impact of sensor noise.

**Effect of Data Augmentation:** Data augmentation plays a crucial role in improving model robustness. We experimented with different augmentation strategies, including random rotations, scaling, and dropout of sensor data. The results indicate that appropriate augmentation not only improves quantitative metrics such as IoU and Chamfer distance but also enhances the visual quality of the reconstructed surfaces by reducing artifacts in regions with sparse sensor coverage.

**Cross-Dataset Generalization:** One of the significant challenges in 3D reconstruction is ensuring that models trained on synthetic or curated datasets generalize well to real-world data. Our experiments on ModelNet40, where the model was trained on ShapeNet and then directly applied to unseen categories, demonstrated promising generalization. Although the IoU and Chamfer distance values on ModelNet40 were slightly lower than those on ShapeNet, the overall reconstruction quality remained high, validating the versatility of our learned implicit representation.

**Comparison with Recent Methods:** We also compared our method with several recent state-of-the-art approaches such as DeepSDF [3], Occupancy Networks [4], and NeuS [8]. Although these methods exhibit competitive performance on certain benchmarks, our multi-sensor fusion framework consistently achieved higher scores on IoU and normal consistency, particularly in scenarios where occlusions and sensor noise were prevalent. For example, in one experiment on ShapeNet chairs, our method achieved an IoU of 0.852 compared to 0.835 for DeepSDF and 0.840 for Occupancy Networks.

**Computational Efficiency:** Despite the high-dimensional nature of the problem, our network is designed for efficiency. By leveraging a compact latent space and efficient feature fusion, the inference time per object was reduced to a few seconds on a modern GPU. We also analyzed the scalability of our method with respect to the number of sensors and spatial resolution. Our experiments showed that while the computational cost increased linearly with the number of sensors, the benefits in reconstruction quality justify the added expense. Furthermore, advanced techniques such as octree-based sampling can be integrated in future work to further optimize performance for large-scale scenes.

**Ablation on Loss Components:** In additional experiments, we varied the weights of the loss components in Equation (9). When the weight λ for the Eikonal loss was set too high, the network overemphasized the gradient constraint, leading to overly smooth reconstructions that lacked fine details. Conversely, if λ was too low, the SDF predictions deviated from the ideal unit gradient property, resulting in artifacts. Our experiments suggest that a balanced setting (with λ typically in the range [0.1, 0.2]) yields the best trade-off between data fidelity and regularization.

### 4.9. Discussion of Extracted Experimental Data from the Literature

To further contextualize our results, we extracted and compared experimental data from several recent studies on 3D reconstruction using neural implicit representations. For example, in the work of Park et al. [3], DeepSDF was reported to achieve an average Chamfer distance of approximately 1.2 × 10^−3^ on a subset of ShapeNet, while our method achieved 1.11 × 10^−3^. Similarly, Occupancy Networks [4] reported IoU values in the range of 0.83 to 0.84, whereas our multi-sensor method attained an IoU of 0.852. These comparisons, extracted from multiple peer-reviewed publications and technical reports, underscore the competitive performance of our approach in both qualitative and quantitative terms.

Moreover, additional studies on TSDF fusion methods report that traditional volumetric fusion methods are prone to noise and discontinuities when dealing with occlusions. Our experiments confirm these findings, as the TSDF fusion baseline consistently lagged behind our method in terms of both IoU and surface smoothness. These insights from the literature provide a solid benchmark against which our method’s performance can be measured.

### 4.10. Summary and Concluding Remarks on Experiments

In summary, our extensive experiments demonstrate that:

- The integration of multiple sensor modalities significantly enhances the completeness and accuracy of the reconstructed surfaces.
- Our method consistently outperformed both single-sensor implicit approaches and classical TSDF fusion techniques across a range of metrics, including IoU, Chamfer distance, normal consistency, and F-score.
- The training process converges steadily, with well-balanced loss components ensuring that the learned SDF maintains both high fidelity to the ground-truth and adherence to the unit gradient constraint.
- Ablation studies confirm the positive impact of multi-sensor fusion and provide valuable insights into the optimal settings for latent dimension, network depth, and loss weighting.
- Qualitative comparisons further validate the robustness and visual quality of our reconstructions, particularly in challenging scenarios with occlusions and sensor noise.

These experimental findings, corroborated by comparisons with recent literature, firmly establish the efficacy of our multi-sensor implicit reconstruction framework. Our results not only demonstrate superior quantitative performance but also highlight the practical advantages of leveraging learned shape priors and effective feature fusion for high-quality 3D reconstruction.

## 5. Discussion

In this section, we analyze the advantages of our multi-sensor implicit reconstruction method, discuss its limitations, and provide insights into its deeper mathematical underpinnings. We also propose several directions for future research.

### 5.1. Advantages and Insights

Our experimental results and theoretical analysis suggest several key advantages of the proposed method:

Enhanced Occlusion Handling. One of the primary benefits of integrating multiple sensor modalities is the improved handling of occlusions. When one sensor fails to capture a particular region due to line-of-sight issues, another sensor, located at a different viewpoint, can compensate. This results in a more complete latent representation z, which, in turn, leads to reconstructions with fewer missing parts.

Leveraging Learned Shape Priors. Even in regions where sensor data are sparse or entirely missing, the network is capable of generating plausible completions. This is achieved by learning statistical priors over the training data, which encode common object shapes and symmetries. Consequently, the network is able to infer the geometry of occluded or unobserved areas, resulting in reconstructions that are both complete and consistent with typical object structures.

Smooth and Watertight Surfaces. The implicit representation based on SDFs naturally produces smooth and watertight surfaces. Unlike discrete volumetric methods, which may result in jagged or fragmented outputs, our approach yields continuous surfaces with well-behaved topology. The incorporation of the Eikonal regularization further ensures that the distance field varies smoothly, preventing artifacts such as flattened or inflated regions.

Robust Feature-Level Fusion. By fusing features at an intermediate level (i.e., after sensor-specific encoding), our method is more robust to the different noise characteristics and resolution limitations inherent in each sensor modality. This feature-level fusion allows the network to weigh the reliability of different sensors dynamically and extract complementary information effectively.

### 5.2. Limitations

Despite its strengths, our method does have some limitations that are important to address in future work:

Dependence on Sensor Calibration. Our approach assumes that all sensors are well-calibrated with accurate intrinsic and extrinsic parameters. Any errors in calibration can lead to misalignment in the global coordinate system, which in turn degrades the quality of the fused representation and the subsequent reconstruction.

Static Scene Assumption. The current implementation is designed for static scenes. Dynamic objects or deformable surfaces present additional challenges that are not directly handled by our method. Future work should consider temporal consistency and motion modeling to extend the approach to dynamic environments.

Computational Cost. While the implicit decoder is efficient, evaluating the SDF over a dense 3D grid remains computationally expensive for large-scale scenes. Techniques such as octree-based sampling or adaptive query strategies could be employed to mitigate this limitation and enable real-time reconstruction for larger environments.

Reliance on Training Data. The performance of our method is heavily dependent on the quality and diversity of the training dataset. If the training data do not adequately capture the variability in object shapes or sensor noise, the model’s generalization to novel or out-of-distribution objects may be compromised.

### 5.3. Deeper Mathematical Implications: SDF as a PDE Problem

From a theoretical perspective, learning an SDF with an Eikonal regularizer can be interpreted as solving a partial differential equation (PDE) of the following form:

(11) $$||\nabla d(x)||=1,\;d(x){|}_{x\in \partial Ω}=0.$$

This PDE formulation offers valuable insights into the behavior of the SDF. By enforcing the unit norm condition on the gradient, the network is encouraged to produce a distance field that increases linearly in the normal direction away from the surface. Such a constraint is not only physically meaningful but also instrumental in preventing pathological solutions in regions with limited data.

A Taylor series expansion of d(x) around a point x0 provides further insight:

(12) $$d\left(x\right)\approx d\left(x_{0}\right)+\nabla d\left(x_{0}\right)\ldots \left(x-x_{0}\right)+\frac{1}{2}(x-x_{0}{)}^{⊤}H(x_{0})(x-x_{0})+\ldots ,$$

where H(x0) is the Hessian matrix of second-order partial derivatives. In the vicinity of the surface, the linear term dominates if the gradient norm is maintained at one. This analysis explains why enforcing the Eikonal condition is critical for obtaining accurate surface representations.

### 5.4. Future Research Directions

Several avenues for future research emerge from our work:

- **Robust Calibration and Dynamic Fusion:** Investigate methods for online sensor calibration and dynamic fusion that can adapt to changes in sensor pose or scene dynamics.
- **Efficient Sampling Strategies:** Develop adaptive grid sampling methods, such as octree or multi-resolution strategies, to reduce computational overhead during surface extraction.
- **Hybrid Approaches:** Explore the integration of classical PDE solvers with neural implicit representations to enforce global consistency and improve the fidelity of reconstructions.
- **Extended Modalities:** Incorporate additional sensor modalities (e.g., thermal imaging or radar) to enhance the robustness and versatility of the reconstruction framework in challenging environments.
- **Temporal Consistency for Dynamic Scenes:** Extend the framework to handle dynamic scenes by incorporating temporal coherence and motion estimation techniques.

In summary, while our multi-sensor implicit reconstruction method demonstrates significant improvements over traditional approaches, addressing the aforementioned limitations will be key to further advancing the state of the art in 3D reconstruction.

### 5.5. Future Work

There are multiple avenues for extension:

- **Adaptive/Hierarchical Sampling.** Replacing uniform 3D grids with octree or GPU-based raycasting methods can accelerate surface extraction, especially for large scenes.
- **Dynamic Scenes.** Extending the method to handle moving objects or scenes over time would require both temporal fusion and robust correspondences between frames.
- **Uncertainty Estimation.** In real-world scenarios, sensor data often have varying noise levels. Incorporating uncertainty (e.g., weighting each sensor’s contribution) could improve reconstruction robustness.
- **Incremental Learning.** Instead of offline training on synthetic data, an online approach could continuously refine the SDF as more sensor data stream in, potentially enabling real-time robotics applications.
- **Combining with Neural Rendering.** Bridging implicit geometry with neural radiance fields or inverse rendering frameworks [8,9] might yield synergy in refining both geometry and appearance from multi-sensor cues.

## 6. Conclusions

We presented a deep implicit surface reconstruction system that explicitly fuses multi-sensor feature embeddings to achieve complete, accurate 3D reconstructions in complex real-world scenes. By fusing encoders specialized for different sensor modalities, our method generates a single unified latent representation feeding into a signed distance function (SDF) decoder. Eikonal regularization ensures consistent gradient norms, improving the surface geometry.

Extensive experiments on ShapeNet demonstrate that multi-sensor fusion significantly boosts performance over single-sensor or classical TSDF baselines. Ablations confirm that adding additional sensors reduces occlusions, leading to better coverage and more faithful reconstructions. Moreover, normal consistency analyses and visualizations support the claim that multi-sensor implicit fusion yields smoother, more coherent surfaces.

Notwithstanding its advantages, the method’s reliance on calibration, static scenes, and offline training highlights areas for improvement. We believe integrating robust sensor uncertainty modeling, dynamic scene support, and more efficient sampling strategies could further expand the applicability of deep implicit multi-sensor reconstruction. Overall, our work underscores the promise of combining multi-view or multi-modal data with learned implicit surfaces, offering a practical route to robust and high-fidelity 3D models across diverse real-world scenarios.

## Figures and Tables

**Table 1:** Computational complexity comparison on ShapeNet chairs.

Method	Inference Time (s) ↓	GPU Memory (GB) ↓
Single-sensor implicit	3.2 ± 0.3	6.5 ± 0.2
TSDF fusion	4.2 ± 0.4	9.8 ± 0.3
DeepSDF	3.8 ± 0.3	8.2 ± 0.2
Occupancy Networks	4.0 ± 0.4	8.5 ± 0.3
**Ours (multi-sensor)**	**2.5 ± 0.2**	**8.0 ± 0.2**

**Table 2:** Quantitative results on a subset of ShapeNet. Our multi-sensor implicit model achieves higher IoU and normal consistency and lower Chamfer distance.

Method	IoU ↑	Chamfer (10^−3^) ↓	Normal Consistency ↑
Single-sensor	0.780	1.53	0.88
TSDF fusion	0.795	1.35	0.89
**Ours (multi-sensor)**	**0.852**	**1.11**	**0.93**

## Data Availability

All data are included in the article.
